# A Rare Case of Solitary Kidney Metastasis Following Primary Laryngeal Squamous Cell Carcinoma

**DOI:** 10.15586/jkcvhl.2017.68

**Published:** 2017-05-03

**Authors:** Sharon Del Vecchio, Robert Ellis, Kylie Gallagher, Keng Lim Ng, Li Ma, Geoffrey Strutton, Simon Wood

**Affiliations:** 1 Faculty of Medicine, Translational Research Institute, University of Queensland, Woolloongabba, Australia; 2 Department of Urology, Princess Alexandra Hospital, Woolloongabba, Australia; 3 Pathology Queensland, Princess Alexandra Hospital, Woolloongabba, Australia

**Keywords:** laryngeal squamous cell carcinoma, PET-MRI, renal cell carcinoma, smoking, solitary kidney metastasis

## Abstract

Laryngeal cancer is the 14th most common malignancy worldwide, and its common subtype squamous cell carcinoma (SCC) is highly associated with tobacco use and long-term alcohol consumption. The incidence of distant metastasis from a primary laryngeal cancer has been reported to be very low, between 6.5% and 8.5%, according to published tumour registry data. Distant metastases of laryngeal SCC most commonly involve the lung, liver, bone and mediastinum, seldom involving the kidney. Renal metastasis has been well established in many other cancers such as lymphoma, lung, breast and gastric carcinoma. This report discusses the rare case of a solitary renal metastasis following a primary laryngeal SCC.

## Introduction

Laryngeal cancer is the 14th most common malignancy worldwide, and its common subtype squamous cell carcinoma (SCC) is highly associated with tobacco use and long-term alcohol consumption (1). The incidence of distant metastasis from a primary laryngeal cancer has been reported to be very low, between 6.5% and 8.5%, according to published tumour registry data (2–4). Distant metastases of laryngeal SCC most commonly involve the lung, liver, bone and mediastinum, seldom involving the kidney (2–4). Renal metastasis has been well established in many other cancers such as lymphoma, lung, breast and gastric carcinoma (5). This report discusses the rare case of a solitary renal metastasis following a primary laryngeal SCC. To the best of our knowledge, this is the fourth report of a primary laryngeal SCC with a metastatic pattern to the kidney and is the first known report of an isolated renal metastasis from a primary laryngeal SCC (6–8).

## Case Report

In May 2013, the patient, a 63-year-old male retired truck driver presented with hoarseness of voice. He is an ex-smoker with a 30-pack year history and history of heavy alcohol use. Other medical history included depression, gastro-oesophageal reflux disease (GORD), hypothyroidism and chronic obstructive pulmonary disease (COPD). Investigations demonstrated a left-sided glottic tumour and the patient proceeded with vertical hemilaryngectomy. Histopathology of the glottic tumour revealed a T3N1M0 SCC with micrometastatic involvement in 1 of the 25 resected lymph nodes. At the time of diagnosis, a positron emission tomography (PET) scan demonstrated no metastatic involvement. Following surgery, the patient attended annual reviews by his ear, nose and throat surgeon and did not demonstrate evidence of recurrence.

He re-presented 42 months later with a 1-week history of right lower quadrant pain and no other associated symptoms. Initial abdominal ultrasound demonstrated no abnormalities and he progressed to contrast enhanced computed tomography (CT) of the abdomen and pelvis. CT demonstrated a focal abnormality involving the pelvic-ureteric junction characterised by mural thickening and hyper-enhancement of the right renal hilum suggestive of a neoplastic process. The lower pole of the ipsilateral kidney also demonstrated a 3 cm cyst that appeared to be benign. The contralateral kidney and liver were unremarkable on CT.

**Figure 1:**
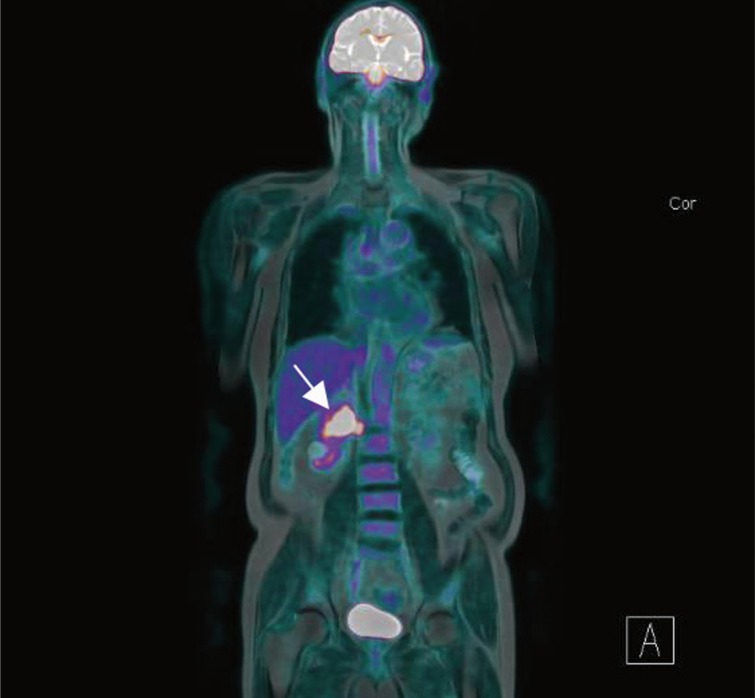
Coronal PET-MRI scan demonstrating a solitary enhancing lesion in the right renal hilum (arrow).

He was referred to a urology team on the suspicion of urothelial carcinoma. The urology team performed right-sided cystoscopy and retrograde pyelogram with lavage cytology which demonstrated a normal collecting system and normal urothelial cells which made the differential diagnosis of urothelial malignancy unlikely. The differential diagnosis included renal cell carcinoma (RCC). He had no haematuria; urine microscopy, culture and sensitivity were normal, and there were no other urological symptoms. The opinion of the interventional radiologist was that a percutaneous biopsy of the mass was too risky given its proximity to major vasculature structures. The patient underwent a positron emission tomography–magnetic resonance imaging (PET-MRI) scan that demonstrated a right-sided renal hilar mass encasing the renal vessels and showed evidence of invasion into the inferior vena cava (IVC) with possible invasion of the right crus of the diaphragm. No metastatic lesions were detected at other sites (Figure 1).

Laboratory tests revealed renal insufficiency with a serum creatinine of 130 µmol/L and an estimated glomerular filtration rate of 49 mL/min/1.73 m^2^ and a urinary albumin to creatinine ratio of 0.9 mg/mmol. The unusual finding of an infiltrative renal hilar mass on CT that did not demonstrate the usual characteristics of RCC on imaging and was not amenable to biopsy was brought to a multidisciplinary team (MDT) meeting for discussion. The MDT came to the consensus that the renal mass should be managed surgically, as if RCC. The risks and benefits of surgical management were discussed with the patient, and he underwent radical nephrectomy with caval thrombectomy and IVC replacement. The patient recovered from surgery.

**Figure 2:**
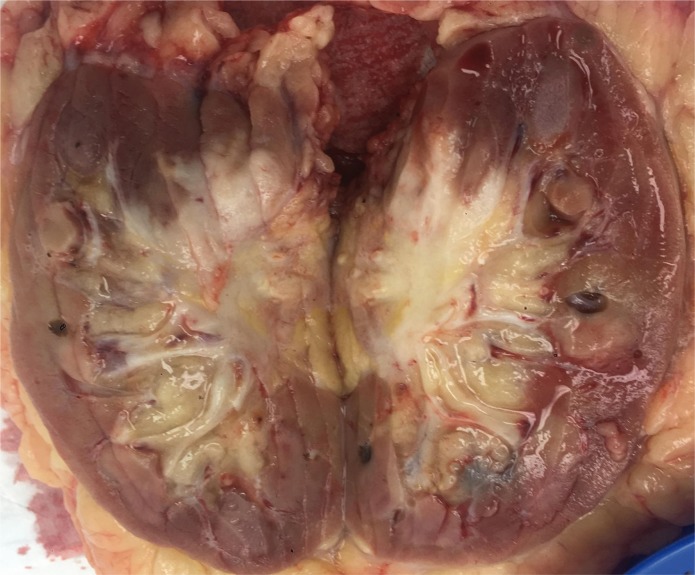
Pathological specimen of right kidney post-nephrectomy demonstrating a dense hilar mass.

**Figure 3:**
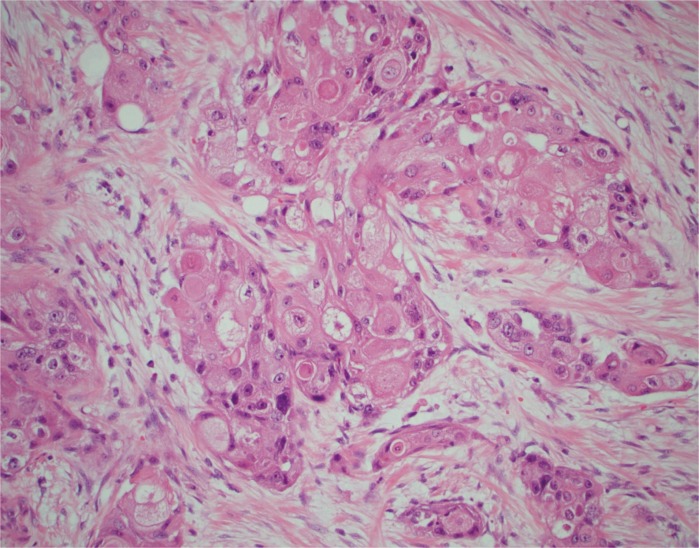
Histopathology from tumour specimen demonstrating a moderately differentiated squamous cell carcinoma with associated fibrosis H&E × 200.

Macroscopic features showed a 45 mm cream-coloured tumour in the mid- to upper pole with no haemorrhage or necrosis (Figure 2). The tumour showed invasion through the IVC wall, extending into the psoas muscle. Histopathology demonstrated a moderately differentiated SCC with associated fibrosis (Figure 3). The tumour also showed extensive lymphovascular invasion and peri-neural invasion involving the vascular margin. Two of the five resected hilar lymph nodes demonstrated involvement, whereas the one resected pre-aortic lymph node did not demonstrate involvement. The patient was referred to a radiation oncologist to discuss the possibility of adjuvant radiotherapy.

## Discussion

This patient underwent nephrectomy for the suspicion of a primary renal tumour that was determined to be an SCC metastasis and not RCC. Had a percutaneous biopsy been deemed safe, an MDT may have treated this patient with neoadjuvant therapy. To the best of our knowledge, there have been three previous reports of primary laryngeal SCC with metastasis patterns to the kidney; however, this is the first report of an isolated renal metastasis.

An earlier report by Paul et al. in 1999 described a 42-year-old male with invasive SCC of the larynx who was managed by total laryngectomy. He re-presented 2 years later with abdominal pain and was found to have a solitary pulmonary nodule in the right lower lobe, as well as a left-sided cystic renal tumour that invaded into the psoas muscle. The patient underwent radical nephrectomy, and histology of the renal mass confirmed moderately differentiated SCC. The patient succumbed 6 months following first evidence of distant metastasis (6).

Lecoeuvre and colleagues reported a 60-year-old male with well-differentiated SCC of the larynx who was treated conservatively with partial laryngectomy. Three years following treatment, he developed local recurrence of SCC and subsequent bilateral pulmonary metastases. He was treated with adjuvant therapy. The following year, a CT demonstrated a mass in the superior pole of the left kidney, which was confirmed to be an SCC on biopsy (7).

Erbag et al. (8) reported a 55-year-old male with SCC of the larynx confirmed on punch biopsy. He was managed by total laryngectomy, bilateral functional neck dissection and a right hemithyroidectomy. Histopathology demonstrated locally advanced metastatic SCC and lymphadenopathy. Five years following the initial diagnosis, a CT showed hypodense masses of the liver and of the right kidney. Partial nephrectomy and metastasectomy of the liver lesion were consistent with SCC.

The prognosis for patients with distant metastases following primary laryngeal SCC has been reported to be poor, with a cure rate of only 4% (4). Certain cancer subtypes may follow organ-specific metastatic patterns, termed the “seed and soil hypothesis” (9). As the pathogenesis of cancer metastasis continues to be elucidated, this rare case may contribute to further understanding of the disease patterns and behaviour of laryngeal SCC.

## Conclusion

This case demonstrates the rare entity of a supra-diaphragmatic primary cancer metastasising to the kidney. Patterns of metastasis from a supra-diaphragmatic primary below the diaphragm are poorly understood and relatively rare; however, when they do occur, they tend to involve multiple organs. This is a rare case of metastatic involvement isolated to the kidney. This report also highlights the importance of an MDT approach for the management of such patients both pre- and post-operatively.

## Conflict of interest

The authors declare no potential conflicts of interest with respect to research, authorship and/or publication of this article.
